# Biological potential of copper complexes: a review

**DOI:** 10.55730/1300-0527.3356

**Published:** 2022-01-04

**Authors:** Jamshaid ASHRAF, Muhammad Asad RIAZ

**Affiliations:** 1 Department of Chemistry, University of Gujrat, Gujrat, Pakistan; 2 Department of Chemistry, Quaid-i-Azam University, Islamabad, Pakistan

**Keywords:** Copper complexes, anticancer, antioxidant, antimicrobial, enzyme inhibition activity

## Abstract

This review comprises the inorganic compounds particularly metal coordinated complexes, as drugs play a relevant role in medicinal chemistry. It has been observed that copper complexes are potentially attractive as medicinal importance. In this review, the most remarkable achievements of copper complexes undertaken over the past few decades as antimicrobial, antioxidant, enzyme inhibition activity, and anti-cancer agents are discussed. This work was motivated by the observation that no comprehensive surveys of the diversity of biological activities of copper complexes were available in the literature.

## 1. Introduction

The uses and applications of metals and their complexes are gaining importance in medicinal chemistry [ 1–3]. The inorganic chemistry is further divided into two categories at medicinal ground: in the first category, ligands alone or protein-bound form are used as medicine; in the second category, metal-based drugs and imaging agents are used in the cases where the central metal ion is responsible to carry out the mechanism of action [4]. Different studies have shown the deployment of transition metal complexes to treat various human diseases. Transition metal complexes show variable oxidation state due to uncompleted d-subshell and, hence, interact with a number of negatively charged ions. This action of transition metal complexes may offer therapeutic opportunities and pharmacological applications [5]. The impact of metals in the coordination compounds is different from nonmetals on human beings. These complexes have a great diversity in their mode of action [6]. These metals have distinct and exclusive physical, chemical, and biological properties due to partly filled d-orbital. Despite all transition metals, copper has gained much attention due to its enormous biological potential [7–9]. For a very long time, coordination compounds have been used to treat cancer and malarial diseases. However, metal-based antibacterial drugs gained not much attention by researchers [10, 11 ]. Hence, it is very key area to research and develop new metal based antibacterial drugs, which could overcome the development of medicinal resistance [12]. Although transition elements have been used to prepare metal-based drugs in laboratory. Azo Schiff base metal complexes have exhibited antifungal and antibacterial activities [13]. Furthermore, many transition elements containing azo Schiff base ligands have numerous biological potentials such as therapeutically energetic analgesic properties [14], antibacterial [15], antiviral [16], or antiinflammatory properties [17]. Similarly, dithiocarbamate complexes of the mixed ligands showed antibacterial, antifungal, antialkylation, and anticancer activities. Recent literature survey gives us evidence that dithiocarbamate complexes are now available as diagnostic kit components and radiopharmaceuticals for medical use [18]. Tetradentate Schiff base complexes attach themselves through dinitrogen-dioxygen atoms with metal. Additionally, they possess specific binding sites due to which they can be considered good candidates as DNA secondary structure probes in cancer therapy [19–22]. Another ligand having biological potential, which form complexes of different symmetries with d-block elements, is thiourea [23]. The chemistry of thiazoles and thiosemicarbazones along with their transition metal complexes has attracted the researchers because of their immense pharmacological and antioxidant activities [24–26]. Transition metal complexes have exclusive nature to combine diversely with different ligands leading to discovery of novel drugs [27]. Bipyridyl ligands have ability to combine with copper metal through two nitrogen sites. In one study, most of the 24 hybrid 2-pyridyl- thiazole derivatives were found to have cytotoxic effect on HL-60 human leukemia, HepG2, MCF-7, and NCI-H292 lung cancer cell lines [28].

## 2. Copper chemistry

Copper is the 29th chemical element of the periodic table and belongs to first series of transition elements. Copper chemistry attracts the researchers due to its flexible redox property, geometry, and variable oxidation state. It was found that copper can bind with DNA more frequently than any other divalent cation supporting DNA oxidation [ 29,30 ]. This binding depends upon charge density and size of copper as well as geometry of resulting adduct. Additionally, it was observed that copper protein proceeds through changing the redox potential, thus facilitating electron transfer phenomenon. Copper also promotes several processes in body such as respiration, energy metabolism, protein regulation, etc. [ 31–33]. The various literature surveys reveal that copper compounds have a greater biological potential, as they are anticonvulsive, antibacterial, antiinflammatory, antifungal, and antimicrobial agents [33–37]. An interesting feature of copper compounds is their use in making anticancer drugs, which could be a way alternative to platinum-based drugs because copper compounds are less toxic, and their mechanism of action is comparatively simple than other anticancer drugs [38]. Several pharmacological studies have highlighted that complexes play an interesting role in making variety of anti-tumor drugs, and their diversity also depend upon the ligand attached to copper metal [39]. It was reported that copper complexes containing pyridyl ligand inhibit breast cancer cell lines [40–43]. Literature surveys reveal that various books and research articles have been published on metallodrugs in inorganic chemistry [44–46], especially on different transition metals [47, 48 ]. But in this review, we have explored the important biological applications of copper complexes in drugs and medicines.

### 2.1. Copper complexes as biologically active compounds

#### 2.1.1. Antimicrobial activity

**Figure 1:**
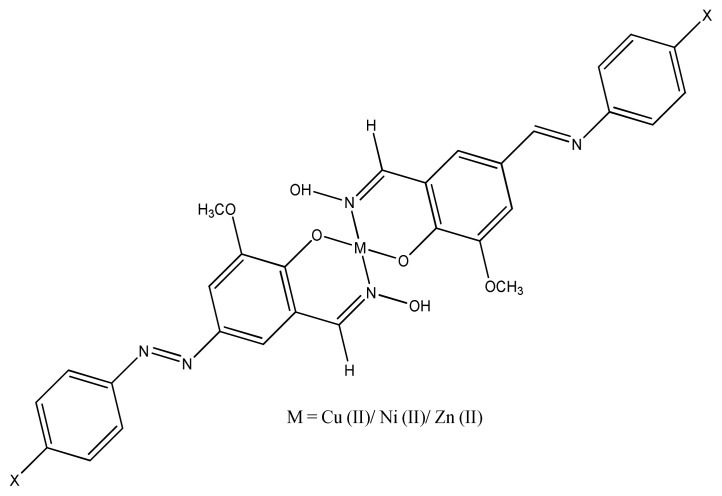
The proposed structure of azo Schiff base complex.

Antibiotics are substances, which either kill or inhibit the reproduction of bacteria and fungi. The treatment of diseases would be impractical today without using antibiotics (Koolman and Roehm, 2005). Binary Cu (II) complexes with a variety of aromatic molecules coordinated through N, S, or O donor atoms have been synthesized and tested for biological activity [ 49–52]. Antimicrobial activity of azo Schiff base ligand and its copper complexes were found out by Slassi et al., and there was no difference in values between ligand and their complexes against three Gram-positive bacteria *E. coli*, *K. pneumonia*, and *P. putida*. The results also indicated that copper complexes have more potent activity than zinc complexes [53–55]. The comparative antimicrobial study of different transition metals with azo Schiff base ligands clearly indicate that copper azo Schiff base complexes (Figure 1) have much better antimicrobial activity than other metal azo Schiff base complexes by using chloramphenicol as positive control. Copper complexes have MIC (minimum inhibitory concentration) values in the range of 15.62–62.5 mM/mL [56, 57 ], which are much better than previous work against Gram-positive (*S. aureus, B. subtilis)* and Gram-negative (*E. coli, S. typhi*) microbes [ 58–60].

**Figure 2:**
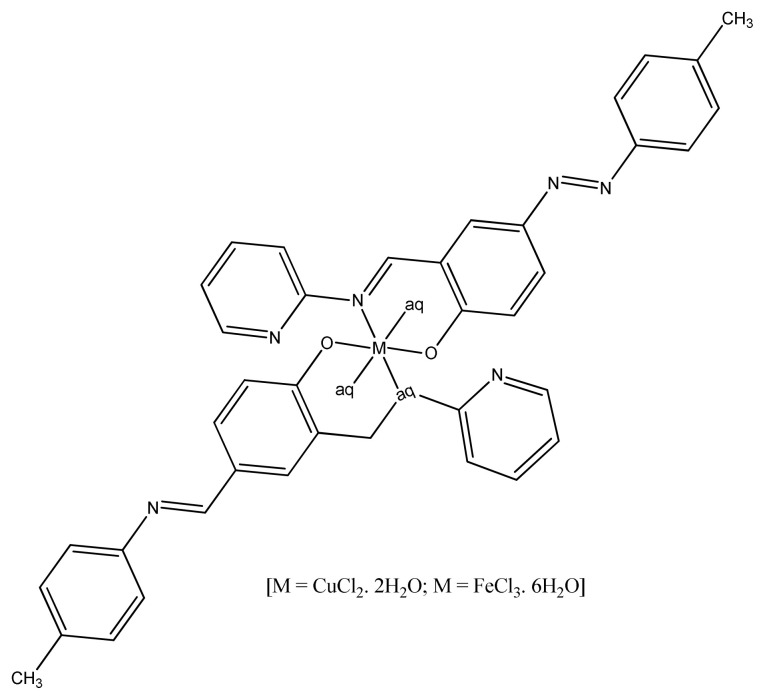
Structure of azo-azomethine complex.

A comparative study of azo-azomethine ligand with complex gave an idea that chelation enhances antimicrobial activity. The Cu (II) and Fe (III) azo-azomethine complexes (Figure 2) exhibited moderate antibacterial activity against *S. aureus and E. coli* strains in comparison with standard antibiotic [61,62 ].

**Figure 3:**
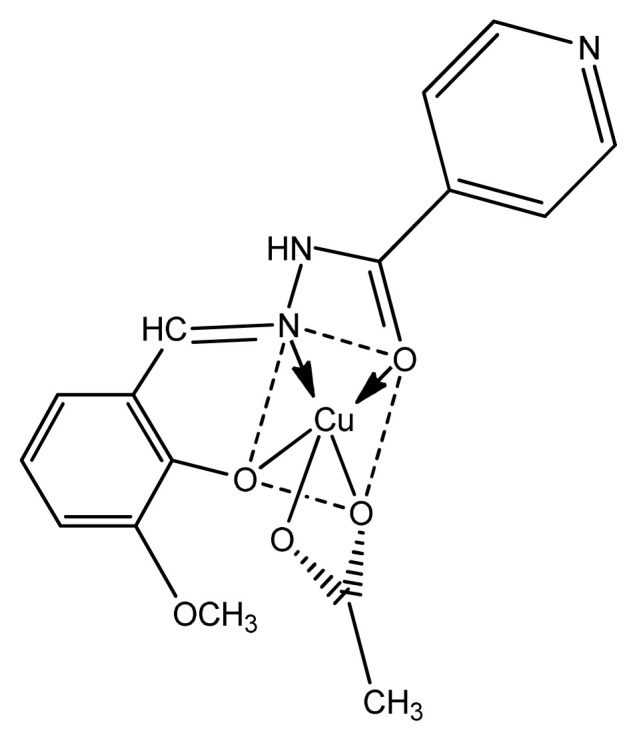
Structure of Cu(II) complex

Another strong evidence comes from the Elena Pahont et al. who showed that azomethine group stimulates antimicrobial activity into complexes due to transformation of mechanisms in biological system. The complexes were screened for antimicrobial activity against Gram-positive (*S. aureus*) and Gram-negative (*E. coli*) microbes and the results were good to best. Furthermore, copper complexes (Figure 3) showed remarkably higher values than other complexes, which is another strong evidence that copper complexes are very good biologically active components [ 63]. Additionally, the data of antifungal activity of these complexes lies in the range of 0.7–250 g/mL against Candida albicans, higher than nystatine activity. The MIC and MBC values of synthesized compounds are greatly affected by nature of metal and the presence of anion within the coordination sphere [64–66].

**Figure 4:**
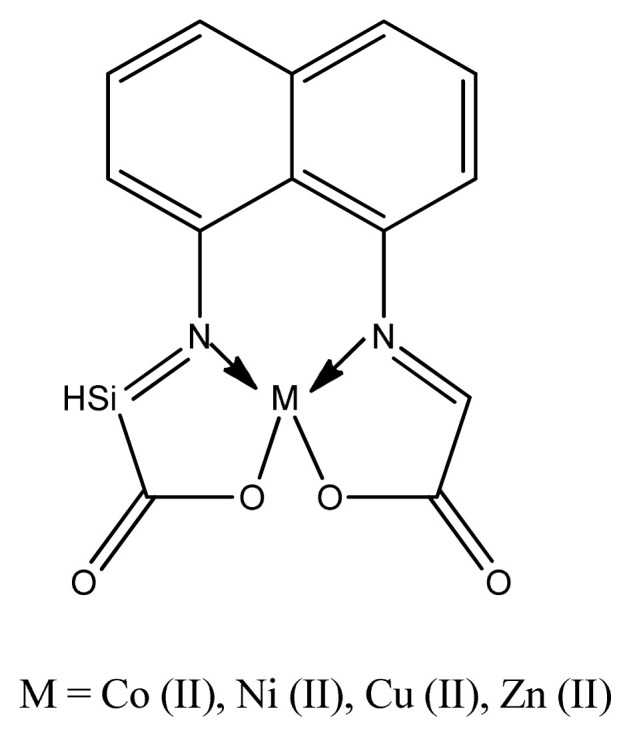
General structure of Schiff base complex.

By using streptomycin as a standard drug, it was observed that copper complexes of oxalate ion and azo anils showed sky-scraping antimicrobial activity than their corresponding ligands against different pathogens like *B. Subtilis, S. aureus, E. coli*, *and E. aerogenes*. Results also tell us that Cu(II) complexes because of having electron withdrawing (azomethine and nitro) groups attached with aromatic system have much antimicrobial activity than all other complexes [67]. This can also be explained nitrogen and oxygen atom attach to anils or Schiff bases also enhances biological activities [68,69 ]. Copper complexes (Figure 4) are very good antimicrobial agent than Zn(II), Ni(II), Co(II) complexes [ 70]. This enhanced inhibiting activity of complexes can be explained in terms of chelation [71,72 ]. Moreover, the coordination of metal with the ligand overcomes the polarity, which, in turn, increases the lipophilic nature of central metal atom or ion. This supports its penetration more proficiently through the lipid layer of microorganisms which destroy them easily [73].

**Figure 5:**
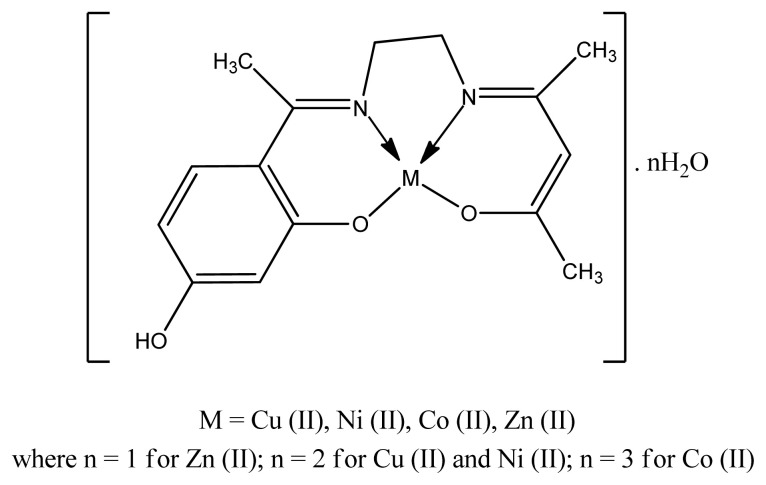
Proposed structure of metal complex.

Balakrishnan et al. prepared nickel (Ni^2+^-K^+^C_5_H_8_NOS_2_^−^) metal complexes and copper (Cu^2+^-K^+^C_5_H_8_NOS_2_^−^) metal complexes. All the complexes and ligands showed good antimicrobial activity toward *B. cereus* MTCC 1272, *S. aureus* MTCC 737, *S. flexeneri* MTCC 1457, and *L. monocytogenes* MTCC 657 microbes. However, their research work indicate that MIC antimicrobial activity values of complexes were less than their ligands against different gram negative and Gram-positive pathogen [74]. The Cu(II), Zn(II) Co(II) and Ni(II) complexes (Figure 5) of (4*E*)-4-[(2-{€-[1-(2,4-dihydroxyphenyl)ethylidene]amino}ethyl)imino]pentan-2-one ligand were synthesized and screened for antimicrobial activity by Ejidike and Ajibade. Among all complexes, copper complex exhibited lower to higher activity against three gram bacteria, namely, *S. faecalis* (ATCC 29212), *S. aureus* (ATCC 25923), and *B. cereus* (ATCC 10702), and three Gram-negative bacteria, namely, *E. coli* (ATCC 25922), *P. aeruginosa* (ATCC 19582) and *S. flexneri* (KZN) than all other metal complexes due to better coordinating ability of metal with the ligand with the order Cu(II) > Co(II) > Ni(II) > Zn(II) > H2LL. This difference in antibacterial activity is due to cell membrane of pathogens and nature of metal atom or ion [75]. Again, resulting complexes are good bactericidal agents than ligands which agrees with Tweedy’s chelation theory and Overtones concept [76].

**Figure 6:**
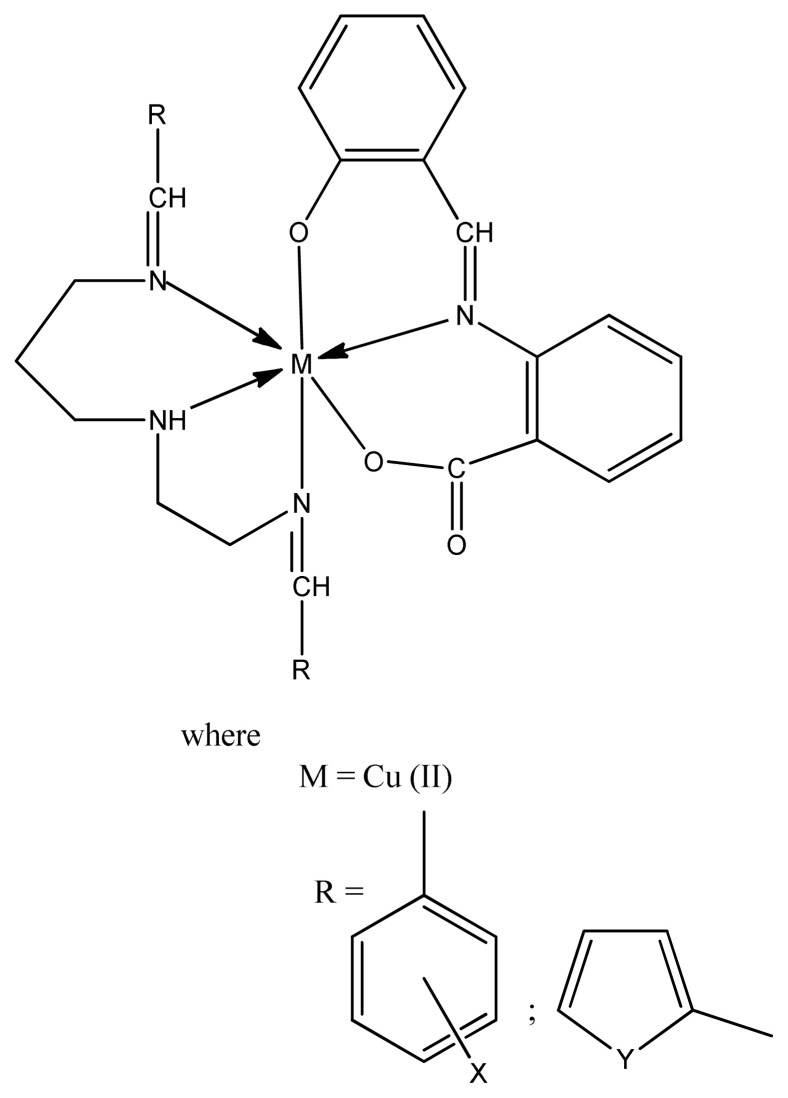
Structure of Cu(II) complex.

The enhanced antimicrobial activity of copper complexes (Figure 6) can also be explained on the basis of size of metal ion, delicacy of particles, solubility, and the presence of bulkiest organic moieties. The better antibacterial activity of [Cu(Lg)(phen)H_2_O]Cl_2_ complex as compared to other copper complexes is due to presence of delocalization of π-charge density within the chelate. Chelation also increases hydrophobic character; thus, it makes easy for complex to enter the cell membrane of pathogen enhancing biological utilization ratio [77].

Chandraleka et al. screened in vitro copper (II) 1,10-phenanthroline and 2,2^’^-bipyridyl Complex for antibacterial activity against S. *typhi, S. paratyphi, E. coli, K. pneumoniae, P. aeruginosa*, and *S. aureus* strains by taking amphotericin B and Ampicillin as a reference drug. The two newly synthesized [Cu(SAla)bpy].H_2_O and [Cu(SAla)Phen].H_2_O complexes showed the highest bactericidal activity against Staphylococcus Aureus, Salmonella paratyphi and Salmonella typhi. These complexes also exhibited antifungal activity in the range of 400–1000 lg/mL [78]. Copper (I) Thiourea and Silver (I) Thiourea showed antimicrobial activity against various pathogens having MIC in the range between 22–34 mm, which is even larger than the standard drug (13.5–29 mm) [79].

**Figure 7:**
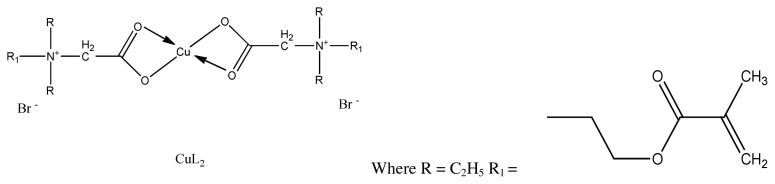
Chemical structure of copper complex.

The nucleophilic character of nitrogen and sulphur atoms and the presence of greater charge into the complex compound facilitate the penetration into the cell wall resulting in the inhibition of their growth. The further study on metal thiourea complexes also gives an idea that increase in polarity of central metal assist the molecules to penetrate more easily into the cell wall of microbe and, thus, deactivate them [80]. Sridhar et al. prepared copper, nickel, cobalt and chromium complexes with (Z)-4-Fluoro-N-(2,7-dimethylhept-6-enylidene) benzenamine ligand and checked their antimicrobial activity in different concentrations for Gram-negative, namely *S. typhi, P. aeruginosa, E. coli*, and three Gram-positive, namely *B. megaterium, S. aures, B. subtilis* microbes by using agar well diffusion method. Almost all complexes exhibited moderate to good activity against gram positive and gram-negative microbes [81]. Among the Cu(II), Co(II), and Fe(III) complexes of nonnatural amino acid ligands, the cobalt complexes showed excellent antimicrobial activity, Cu(II) complexes (Figure 7) showed good and Fe(III) complexes exhibited moderate activity against all the tested strains [82].

Shukla synthesized copper (ii) salicylaldehyde schiff base complex and screened in vitro antimicrobial activity by using agar well diffusion method. [83]. The resulting compounds complexes have higher antimicrobial activity than the ligands which again can be explained with the help of Tweedy’s theory.

Another important feature of coordination chemistry reveals that the geometry of the copper complex is also associated with antibacterial activity. The core of central metal atom determines the electron density in a complex. The copper and cobalt complexes of imidazole derivative ligands having CuN_2_O_2_ geometry tend to exhibit very good antimicrobial activity against different gram positive and Gram-negative strains, while those having CuN_4_ geometry showed no activity. However, both have square planer geometry. The resulting complexes are toxic so cannot be used as medicines [84].

**Figure 8:**
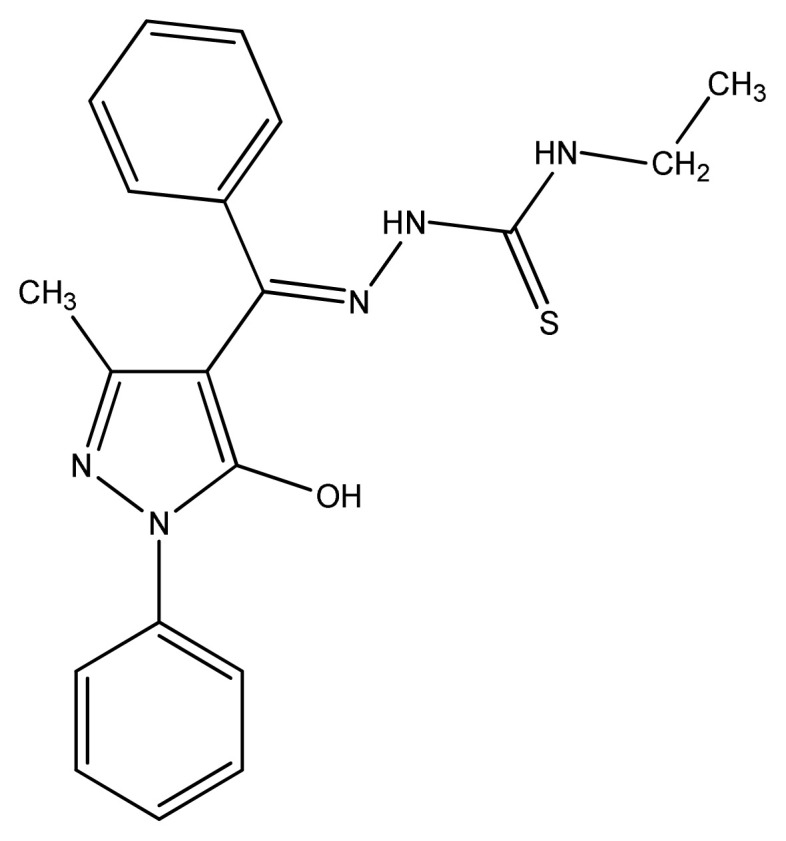
General structure of substituted thiosemicarbazone.

Elena et al. used thiosemicarbazones (Figure 8) to prepare copper and cobalt complexes. The ligands showed no activity, but complexes showed bactericidal and bacteriostatic activity in the range of 1.5–30 lg/mL against Gram-negative and Gram-positive microbes. All copper complexes showed higher antimicrobial activity toward *B. cereus* and *S. aureus* than all other pathogens. The MBC and MIC values are greatly influenced by the presence of copper in the composition of the coordination compounds [85].

**Figure 9:**
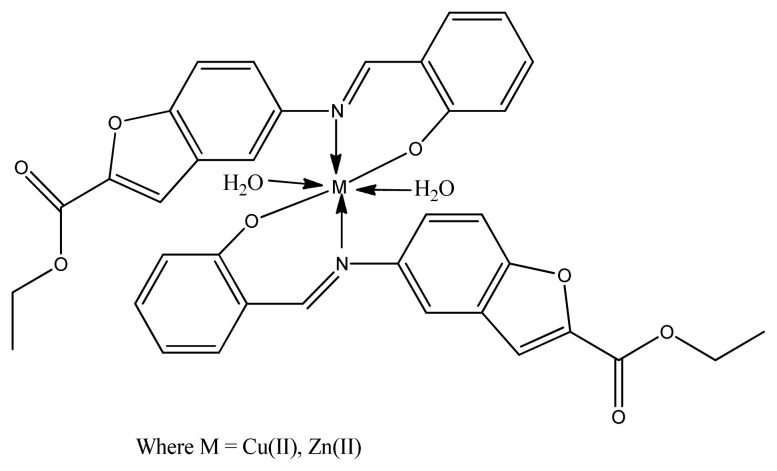
General structure of metal (II) Schiff base complex.

Recently, Zn(II) and Cu(II) complexes (Figure 9) of 5-aminobenzofuran-2-carboxylate Schiff base ligands were synthesized and characterized by Bhushan et al. The results of antimicrobial activity screened by disc diffusion method tell us that all ligands and the coordination compounds are more active than the standard antibiotic. However, copper complexes demonstrated stronger activity than zinc complexes against *E. coli, S. aureus*, and *B. subtilis*. This is maybe due to better coordinating ability of copper (II) metal with biomolecules [86].

**Figure 10:**
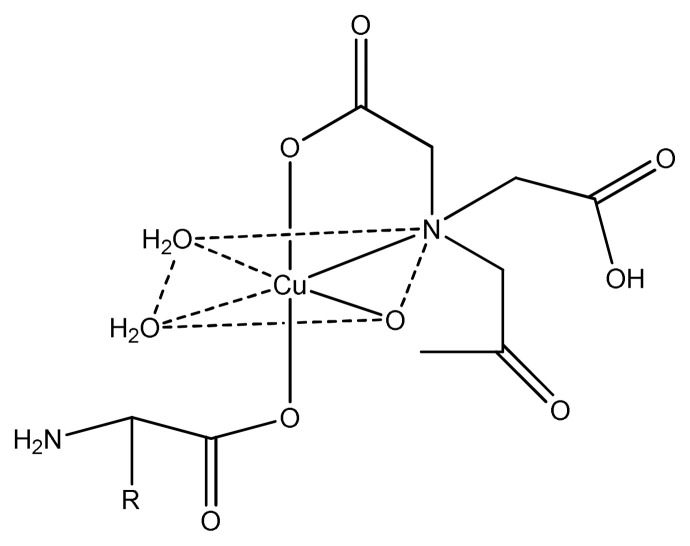
Structure of Cu(II) complex.

Also, the penetration of copper complexes through the cell membrane of pathogens is sky scraping than zinc complexes causing cell death [87]. The minimum antimicrobial activity of nano size copper (II) complexes (Figure 10) was observed against Klebsiella spp. at 0.1 mg mL^−1^, but copper complexes and its ligands exhibited maximum activity towards all other microbes such as Shigellaspp and Samonellae sp. The MIC values tell us that some of copper metal complexes were even more active than reference drug. The copper complexes exhibited more potential than the ligands [88].

**Figure 11:**
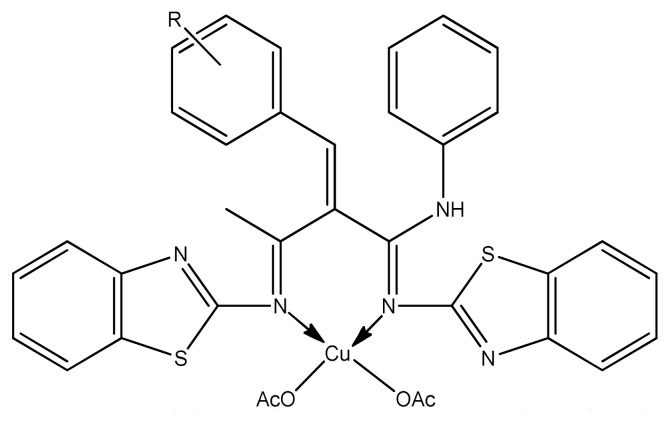
Cu(II) complex containing 2-aminobenzothiazole ligand.

Joseph et al. prepared copper complexes with 2-aminobenzothiazole derivatives by condensation of Knoevenagel reaction. The resulting copper complexes (Figure 11) exhibited greater antimicrobial activity than the ligand against gram positive and Gram-negative microbes. These values showed strong activity than the standard drug Streptomycin. This is due to coordinating ability of two imine groups with the central metal atom which enhances biological activity [ 89].

**Figure 12:**
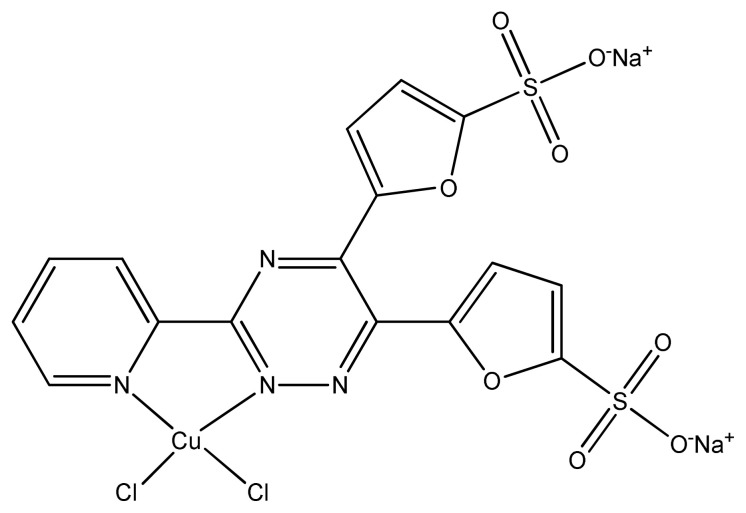
Proposed structure of sulfonated copper-triazine complex.

The sulfonated copper-triazine complexes (Figure 12) did not show significant antifungal and antimicrobial activity in comparison with the standard drug Gentamicin. Two complexes were just less active and other two are almost inactive against *S. aureus* and *E. coli* microbes [90,91 ].

**Figure 13:**
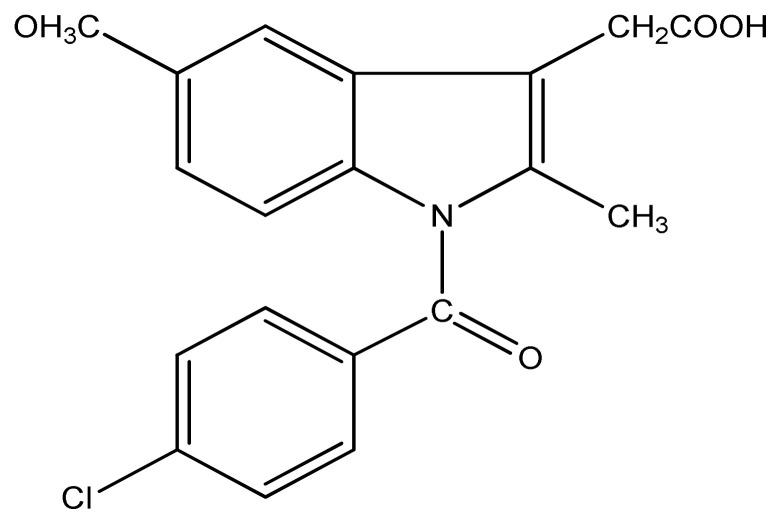
Structure of indomethacin ligand.

Abhijit et al. have shown that manganese, copper, zinc, and cobalt complexes of indomethacin ligand (Figure 13) have moderate to good antimicrobial activity in comparison with the standard drug ciprofloxacin. Among all the complexes, indomethacin manganese complex demonstrated the highest cytotoxicity with the lowest value of LC_50_ 1.222 ± 0.21 μg/ml [ 92]. Janaki and his coworkers prepared curcumin-based copper complexes of 2-aminobenzothiazole derivatives and evaluated them in vitro for antimicrobial activity against *P. aeruginosa, S. aureus, K. pneumaniae*, and *E. coli* bacterial species. Almost all synthesized copper complexes showed good antimicrobial activity against all strains which were even more significant than the ligands [93,94 ].

#### 2.1.2. Antioxidant activity

**Figure 14:**
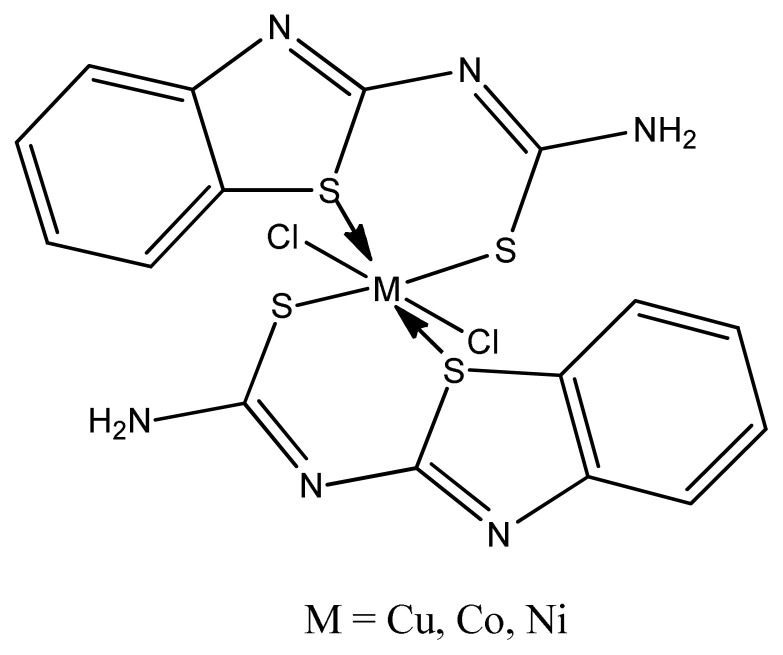
General structure of metal thiourea complex.

Yapati et al. prepared metal (II) complexes (Figure 14) with 1-(benzo[d]thiazol-2-yl) thiourea ligand and checked their free radical scavenging activity by using DPPH method. Almost all compounds showed moderate to high antioxidant activity but copper (II) complexes demonstrated higher scavenging activity than Co (II) and Ni(II) complexes. The IC_50_ values of copper complexes were even comparable with the positive control BHT [95].

**Figure 15:**
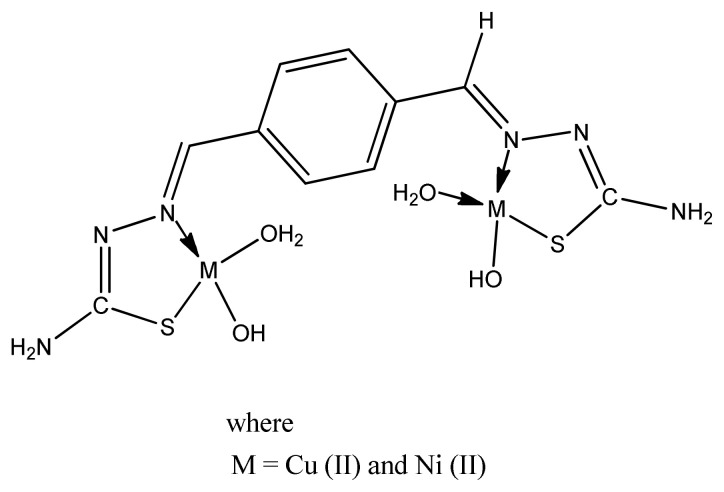
Structure of thiosemicarbazone complex.

Thiosemicarbazone metal complexes (Figure 15) were synthesized and screened for antioxidant activity in vitro by Nitric oxide (NO) scavenging activity, H_2_O_2_ scavenging activity, reducing power and DPPH free radical scavenging method. The results clearly show that copper (II) complexes have high reducing power, H_2_O_2_ Inhibition, nitrous oxide inhibition, and DPPH scavenging values. This detailed study also reveals that chelation of different copper complexes with terepthaladehyde bis-(thiosemicarbazone) greatly affect the antioxidant activity in different models [96].

**Figure 16:**
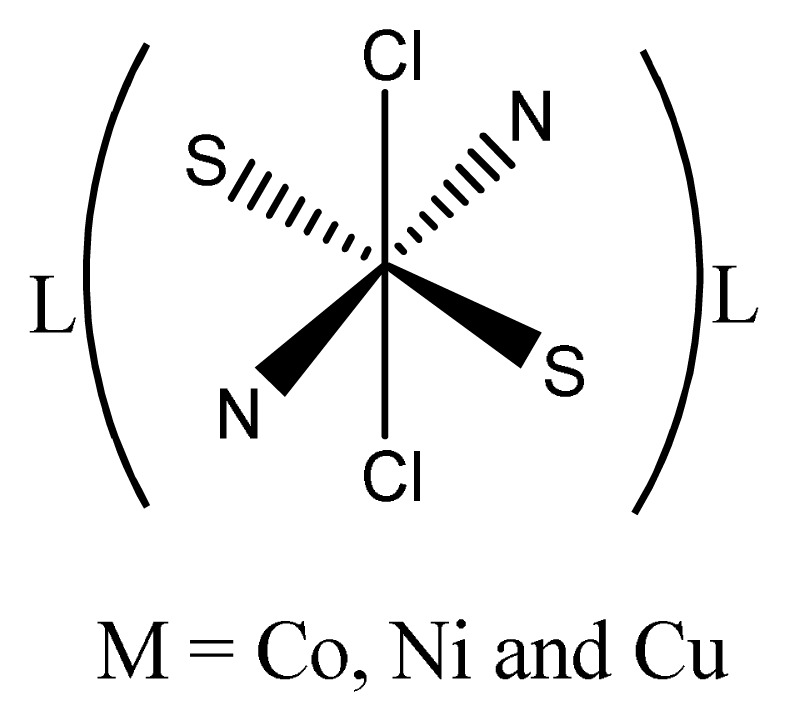
Proposed structure of pyrrolidone thiosemicarbazone complex.

Free radical scavenging activity of metal complexes of Indomethacin ligand was checked by using tert-butyl-1-hydroxytoluene as positive control. Manganese indomethacin exhibited highest antioxidant activity as compared to the other complexes [97]. Copper, cobalt, and nickel ions were used to prepare pyrrolidone thiosemicarbazone complexes (Figure 16). These complexes have very good antioxidant activity in comparison with positive control ascorbic acid. The study also reveals the detailed mechanism of antioxidant activity. The secondary hydrogen atom of amine is prejudiced by inductive effect and allylic double bond. Both factors push electron density toward free radical causing stability of molecule [98].

**Figure 17:**
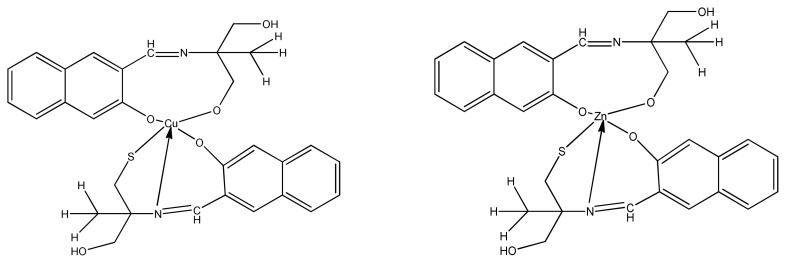
Structure of imine-based complexes.

The IC_50_ values of free radical scavenging activity of complexes indicate that newly synthesized imine-based ligands copper complexes have more pronounced antioxidant activity than imine-based ligands zinc complexes (Figure 17). This also suggests that chelation promotes free radical scavenging [99].

**Figure 18:**
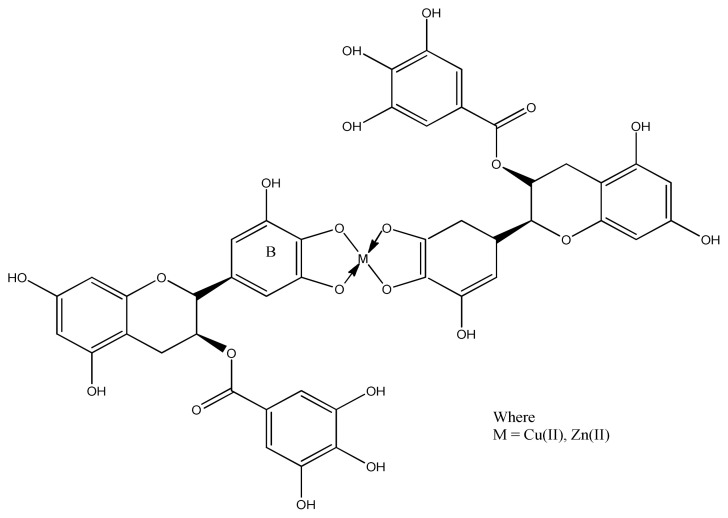
Proposed structure of EGCG metal complex.

A number of literature data reveals that redox properties depend upon axial ligation, size of chelate ring, and extent of double bond in the chelate ring [100]. It has been demonstrated in several papers that chelated metal complexes have significant antioxidant activity than the ligands. This effect can be explained in terms of nature of metal ion as well as chelation effect of imine [101]. Maysoon et al. prepared (−)-epigallocatechin gallate (EGCG) complexes of copper and zinc ions (Figure 18). The experimental results of removing DPPH suggest that there is no difference of antioxidant activity between the EGCG ligand and their complexes [102], may be due to weak interactions between metal ions and polyphenols [103]. These metal ions retain their free radical scavenging activity with EGCG [104].

Curcumin based copper complexes of 2-aminobenzothiazole derivatives showed considerable redox potential in the range from 0 V to –1.6 V, which is possibly due to presence of highly conjugated curcumin analog system having two azomethine groups. The decreasing order of antioxidant activity of copper coordinated compounds is [CuL_2_Cl_2_] > [CuL_1_Cl_2_] > [CuL_4_Cl_2_] > [CuL_10_Cl_2_] > [CuL_8_Cl_2_] > [CuL_7_Cl_2_] > [CuL_5_Cl_2_] > [CuL_9_Cl_2_] > [CuL_3_Cl_2_] > [CuL_6_Cl_2_].

**Figure 19:**
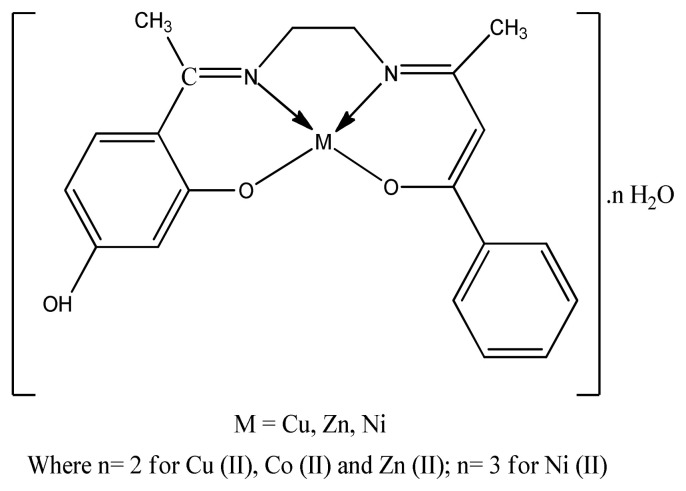
General structure of Schiff base complex.

Ejidike and Ajibade synthesized metal (II) complexes (Figure 19) of (3*E*)-3-[(2-{(*E*)-[1-(2,4-Dihydroxyphenyl) ethylidene] amino} ethyl) imino]-1-phenylbutan-1-one Schiff base ligand and determined in vitro free radical scavenging activity by DPPH method, which is mostly used by researchers because of its simplicity and fastness. The increased antioxidant activity is due to electron withdrawing effect of metals, which makes it easy for DPPH to release proton. This releasing of proton is more pronounced in Cu(DEP) than in Ni(DEP), Zn(DEP) and Co(DEP) complexes. The results suggest that these compounds have a potential to neutralize free radicals, so they can be used for the treatment of pathological diseases [105].

**Figure 20:**
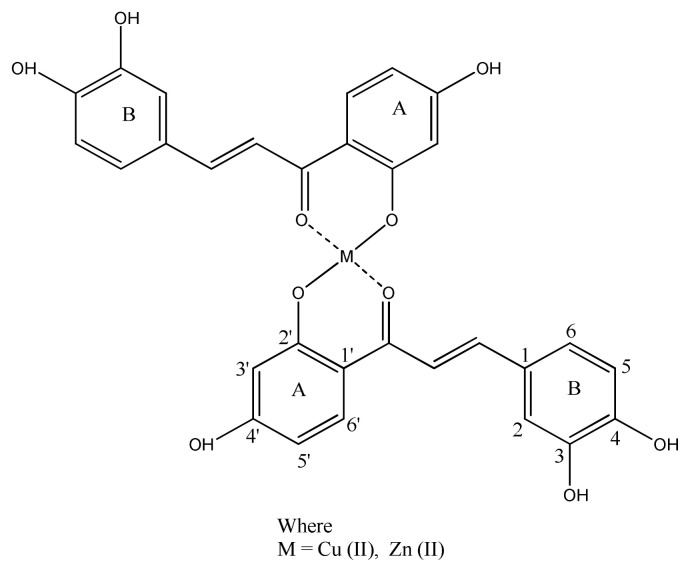
Chemical structure of Metal (II) polyhydroxychalcone complex.

Cu^2+^ and Zn^2+^ coordinated polyhydroxychalcone complexes (Figure 20) were synthesized by Chiara et al. The DPPH method was used to determine antioxidant activity, as it is a nonenzymatic method. The resulted complexes [Zn(ISO)_2_], [Zn(BUT)_2_] and [Cu(ISO)_2_] exhibited higher antioxidant activity than butein and isoliquiritigenin free ligands [106].

**Figure 21:**
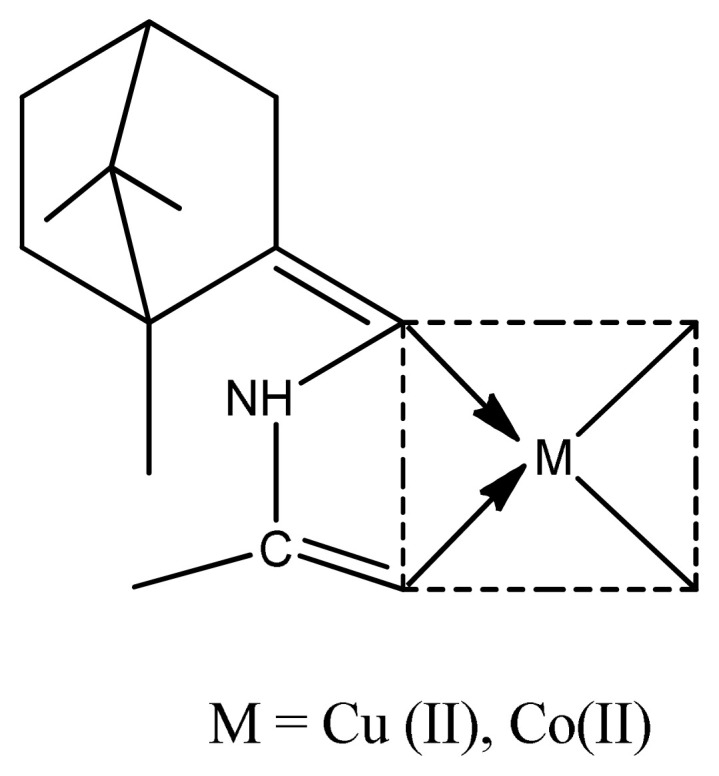
Proposed structure of Metal (II) complex.

DPPH method is mostly used by researchers to determine antioxidant activity because it is a simple, versatile, rapid, and reliable parameter. The IC_50_ value for copper complex (Figure 21) is 111.0 μg/mL, which is far greater than other complexes but less than ascorbic acid (136.0 μg/mL), which were used as reference [107].

In vitro, screening of newly synthesized metal azo-azomethine complexes was done for antioxidant activity by DPPH free radical scavenging method. The IC_50_ clearly indicate that these metal complexes have higher scavenging activity than the previous work [108].

The antioxidant activity of resulting copper complexes is much greater, and it is probably due to presence of hydroxyl group and efficient hydrogen donor to stabilize the unpaired electrons. The introduction of nitro group into the ligand markedly enhances the antioxidant activity of resulting copper complexes. The antimicrobial and antioxidant behavior of copper complexes is in the following order. [CuL^6^(OAc)_2_] < [CuL^1^(OAc)_2_] < [CuL^3^(OAc)_2_] < [CuL^2^(OAc)_2_] < [CuL^4^(OAc)_2_] < [CuL^5^(OAc)_2_].

**Figure 22:**
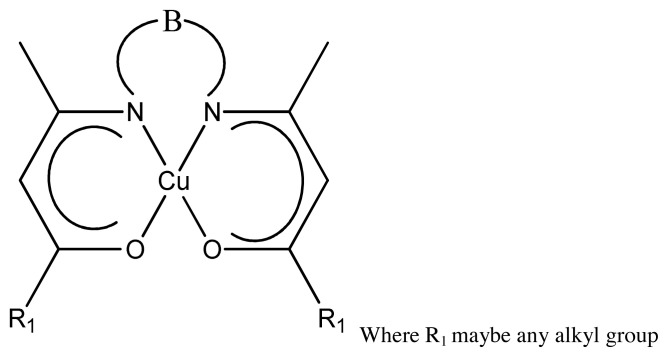
General structure of Copper (II) complex.

Najat et al. attempted to study the mechanism of antioxidant activity. The spin of central metal complexes influences the free radical scavenging activity. The gradual substitution of methyl by phenyl group into the complex increases sum spin density, which, in turn, increases antioxidant activity. If chelate plane angle of a complex is high, then it is difficult for a molecule to transfer the unpaired electron toward DPPH resulting in reduction of antioxidant activity. Furthermore, the Copper (II) complexes (Figure 22) having propylenediamine ligand have significant higher value of scavenging activity than the complex having propylebediamine in bridge form [109].

#### 2.1.3. Anticancer activity

**Figure 23:**
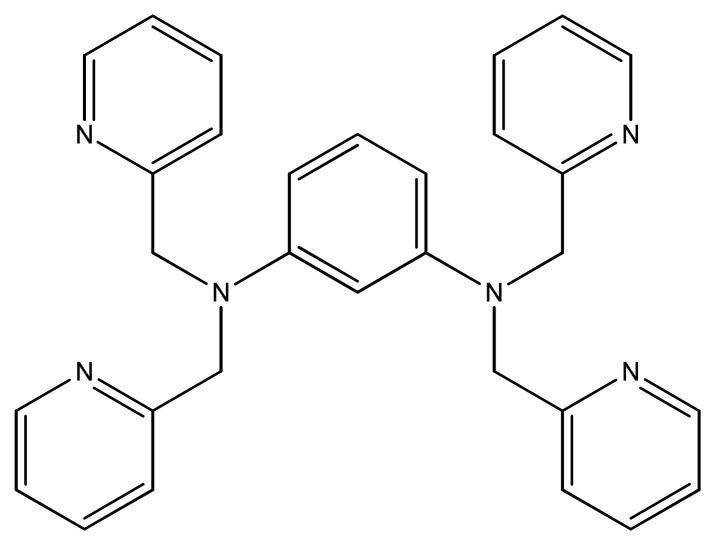
Structure of tripodal ligand.

Melanie et al. prepared and crystallographically characterized copper complexes with tripodal ligands (Figure 23). One of the synthesized compounds was excluded from activity because of having methanol. Among all, one of tetranuclear copper complex [Cu_4_(1,3-tpbd)_2_(SO_4_)_4_] showed promising activity against human solid tumor even 14 times greater than metallodrug cisplatin [110].

**Figure 24:**
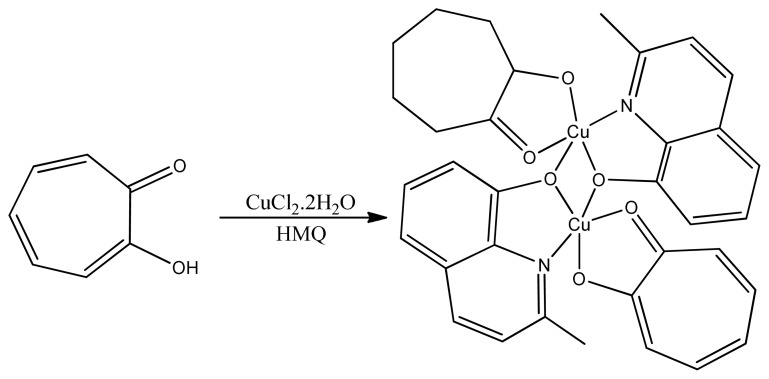
Synthesis of Cu(phen)LCl complex.

Copper (II) complexes containing pyridoxal-semicarbazone ligand were synthesized and characterized as well as being evaluated for anticancer activity. Two out of three copper complexes were slightly active against cancerous cells with cytotoxicity [111]. But, in our research work, two active copper complexes presented less cytotoxicity having IC_50_ values in the range of 50–100 μM toward human breast cancer cell lines. Many copper (II) complexes of Schiff bases were explored as cytotoxic agents as four Cu (II) complexes showed mild cytotoxicity against normal cells and higher against cancerous cell lines [112]. Similarly, many copper chromone complexes have been known to have anticancerous activities [113]. In the same way, four copper (II) complexes with tropolone ligand were synthesized and characterized by Xiyu et al. One ([Cu(phen)LCl].0.5H_2_O) (Figure 24) out of four synthesized complexes was proved to be the best candidate to inhibit cancerous cells, so it might be the anticancer drug even more effective than cisplatin. The present study also reveals that chelated copper complexes have more potency than the ligands [114].

**Figure 25:**
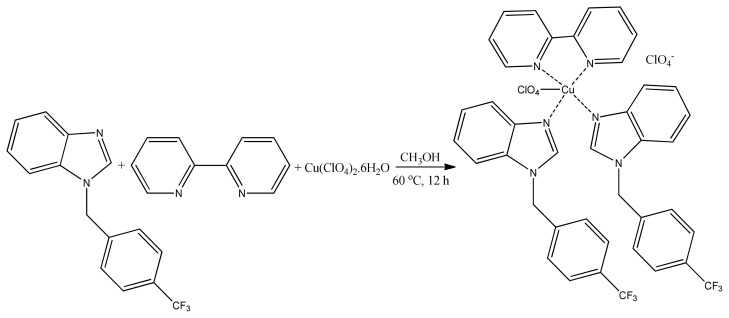
Scheme of synthesis of [Cu(benzimCF_3_)2(bipy)(ClO_4_)](ClO_4_) complex.

Copper is an endogenous metal rather than exogenous. Furthermore, it has considerable DNA and redox potential. In addition, it is easy to synthesize copper complexes [115]. Antiproliferative activity was tested on mononuclear copper (II) complex containing benzimidazole and pyridyl ligands. The MTT results of synthesized copper (II) compounds (Figure 25) demonstrated that they have strong anticancerous effect on DU145 cells [116].

**Figure 26:**
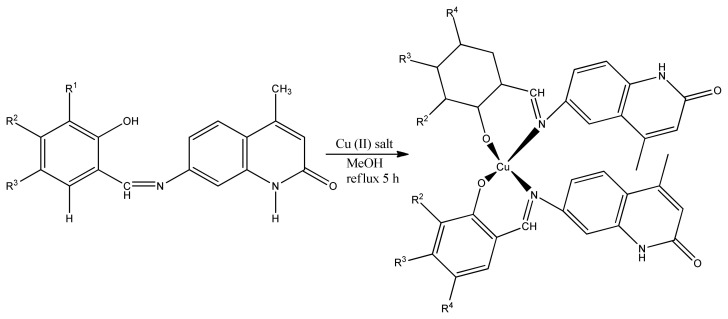
Synthetic scheme of Schiff base Copper (II) complex.

Several copper (II) complexes containing pyridine Schiff base ligand have the ability to inhibit tumor cells [117]. In our present work, we have used MTT assay to study antiproliferative activity. The IC_50_ (39.67 and 44.33.) of two newly synthesized copper complexes (Figure 26) of quinolin-2(1H)-one-derived Schiff bases compounds demonstrate that they have the ability to kill cancerous cells in human body [118].

**Figure 27:**
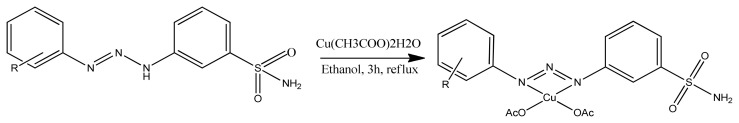
General synthetic route of novel Cu(II) complex.

Recently, sulphonamide based CA inhibitor compounds are in clinical trial for treatment of metastatic breast cancer [ 119]. Similarly, 1,3-diaryltriazene scaffold is one of the most promising and emerging novel compounds, which is used in the growth of anticancer molecules [120]. Novel silver and copper complexes of 1,3-diaryltriazene-substituted sulfonamides (Figure 27) were synthesized and characterized by Dilek et al. among all the tested compounds by MTT assay, the Cu (II) of the type CuL_2_ exhibited greater cytotoxicity having IC_50_ equal to 2.08 μM, which is comparable to standard 5-FU (5-Fluorouracil). One of the silver complex L2-Ag also showed significant antiproliferative activity toward cancer cell lines [121].

Copper(I) complexes of thiosemicarbazones and its derivatives have scholarly attracted the researchers because of its immense role in cancer chemotherapy [122, 123 ]. It has been reported that novel six bis(thiosemicarbazone)copper(I) complexes of the type [CuL_2_Cl] effectively killed EAC (Ehrlich ascites carcinoma) as compared to MCF-7 (human breast adenocarcinoma), HeLa (cervical), and Hep-2(epithelioma) cancer cell lines. The IC_50_ values make us clear that of newly synthesize Cu(I) complexes are less toxic towards normal cell and more toxic against cancerous cells [ 124]. In addition, that IC_50_ values of these newly synthesized compounds have much lower than previously reported thiosemicarbazone-based copper(I) complexes [125, 126 ] and thiosemicarbazone-based copper (II) complexes [ 127, 128 ].

**Figure 28:**
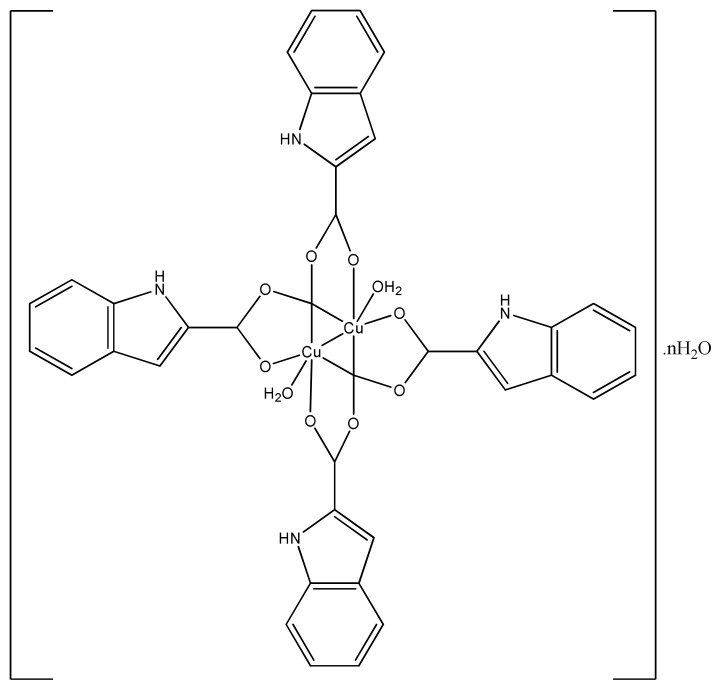
Chemical structure of the Indole-2-carboxylic acid copper complex.

Our previous work showed that cadmium and copper complexes of indole carboxylic acids (ICA-Cu) are very toxic against proliferative cells but less toxic for normal cells [ 129,130 ]. Further it was observed that ICA-Cu has significant inhibitory effect against human breast cancer in a concentration dependent manner. The IC_50_ values of two ICA-Cu molecules were 5.69 μM and 5.43 μM. The results show that these copper complexes (Figure 28) were toxic against two human cancer cells using DMSO as a control [ 131].

**Figure 29:**
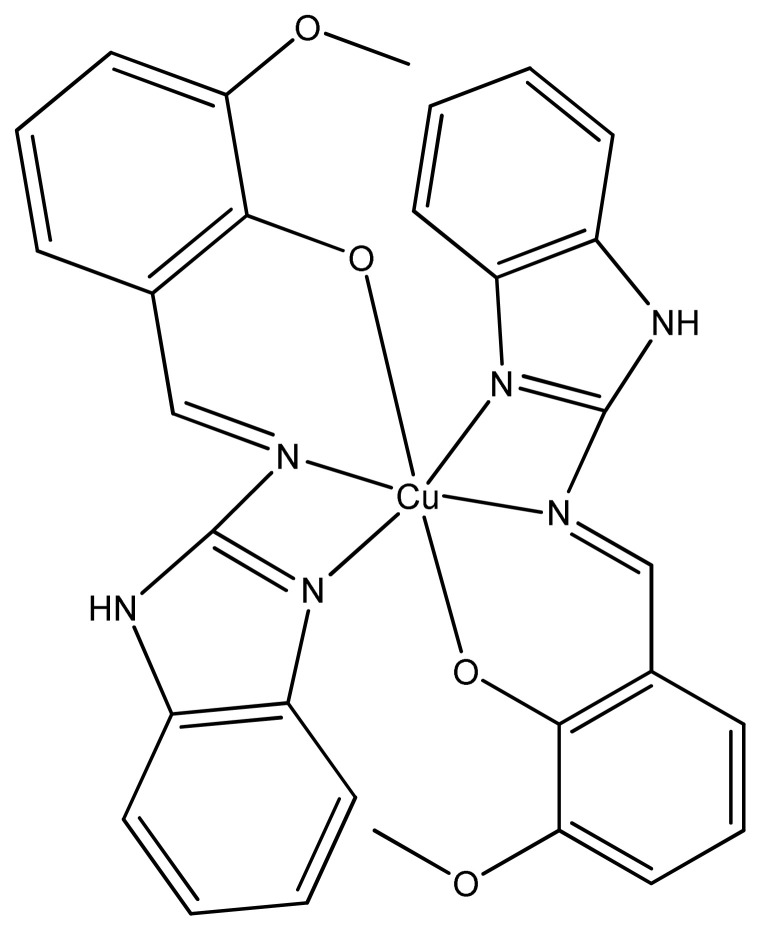
Structure of Cu(II) tridentate Schiff base complex.

The benzimidazole’s derivatives have attracted the researchers studying anticancer, antiviral, and antiinfective activities [132]. In the current study, Cu (II) complexes of tridentate Schiff base ligand were prepared, characterized and studied as antiproliferative agent. All the three copper complexes were known to have inhibitory effect against MCF-7 (human breast cancer) cell lines. In addition, copper metal complexes (Figure 29) exhibited greater anticancer activity in dose dependent manner than heterocyclic moieties in the tested cells. Furthermore, these three copper complexes showed less cytotoxicity toward normal human embryonic kidney (HEK293) [133].

**Figure 30:**
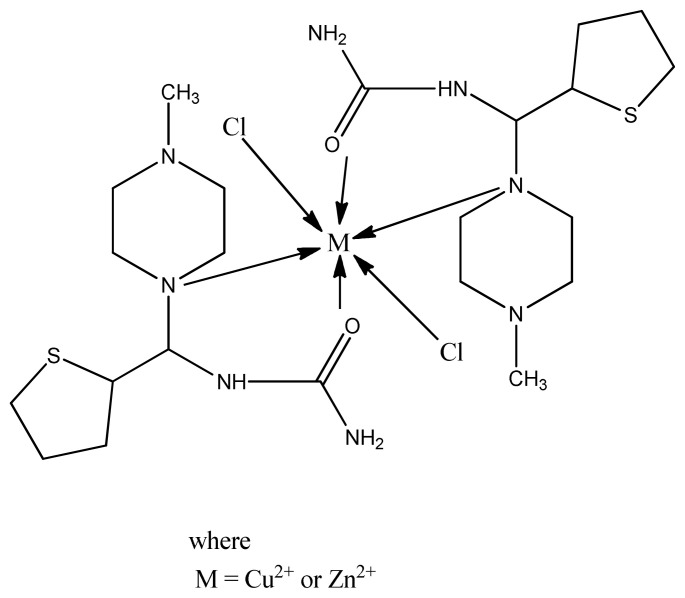
Chemical structure of M(II) complex.

During the cytotoxic study of novel ligand and their complexes, Mohamed et al. synthesized new copper (II) and zinc (II) complexes with the help of mannich bases, which revealed potent antiproliferative activity against colon cancer (IC_50_ = 11.6%) and human lung cancer (IC_50_ = 12.5%) cell lines. In addition, both these compounds (Figure 30) exhibited 55.5% of cell viability of normal cell line (VERO) [134].

**Figure 31:**
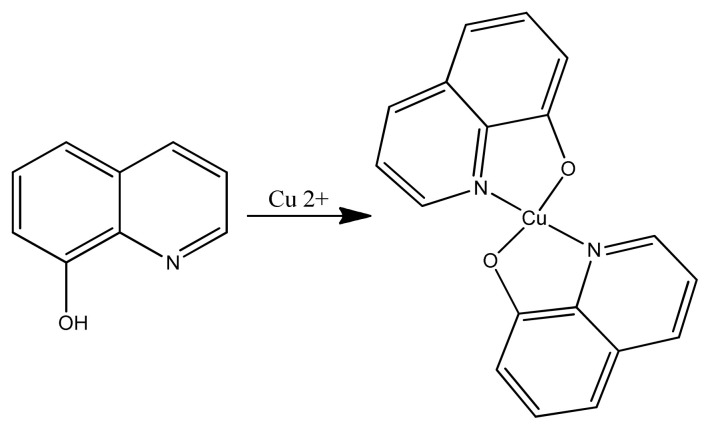
Synthesis of Cu(II) complex.

Diethyldithiocarbamate (DDC) [135], Pyrithione (Pyr) [136], Plumbagin (Plum) [137], 8-hydroquinoline (8-HQ) [138] and Clioquinol (CQ) [139, 140 ] possess individually anti-proliferative activity but the present study relates the anticancerous activity of their copper complexes with Pt resistant drugs. We observed that four out of five ligands exhibited IC_50_ below 10μM against eight cancer cell lines as compared to Pt sensitivity. Furthermore, one copper complex (Cu (DDC)_2_) (Figure 31) caused 50 % reduction in tumor size against Pt-resistant ovarian cancer xenograft [141].

**Figure 32:**
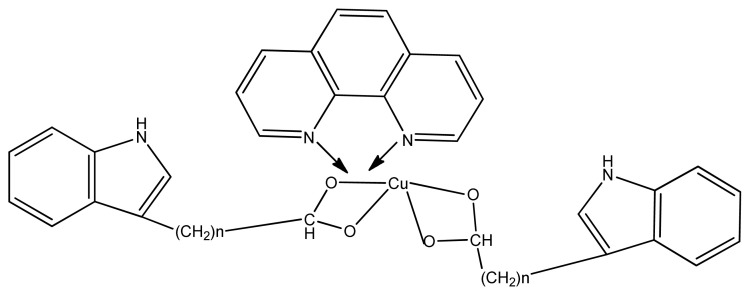
Proposed structure of copper complex.

Zhen et al. observed that copper (II) complexes (Figure 32) with 1,10-phenanthroline and 3-indolecarboxylic acid could enter the tumor cell by developing hydrophobic interaction and hydrogen bonds with the catalytic site of subunit β5 causing chymotrypsin-like activity leading to cancer cell death. The study reveals that copper complexes and other related molecules can act as potent proteasome inhibitors and further be used for the development of anti-cancer agents [ 142].

**Figure 33:**
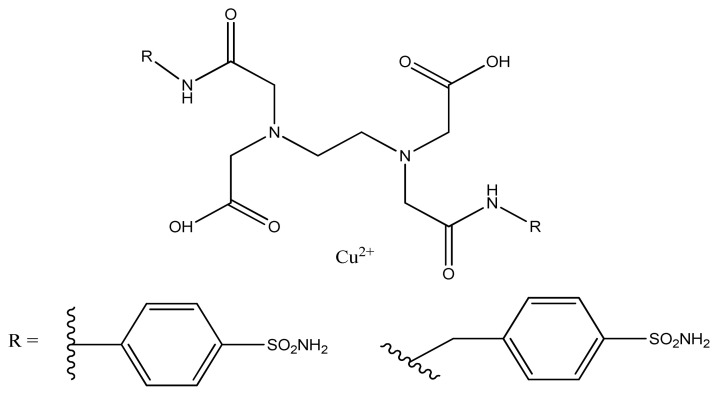
General structure of sulphonamide Cu (II) complex.

It has been recently reported that copper transporters including ATP7B, hCtr1 and ATP7A import subcellular distribution and export of cisplatin-related drugs. This makes us think that copper transporters can legalize the sensitivity of human cancer cells to Pt drugs. These experimental findings are quite appealing because physiologic chemistry of copper and cisplatin is quite different. Even though the mechanisms by which cisplatin drugs act are still unknown, the Cu transporters are tremendously discriminate between closely related metal ions (Figure 33) even between Cu(I) and Cu(II). Thus, it is suggested that variation in platinum drug cellular pharmacology and modified expression of the copper transporters are interceded by secondary effects of Cu on other metabolic pathways, such as GSH and MT levels [143].

**Figure 34:**
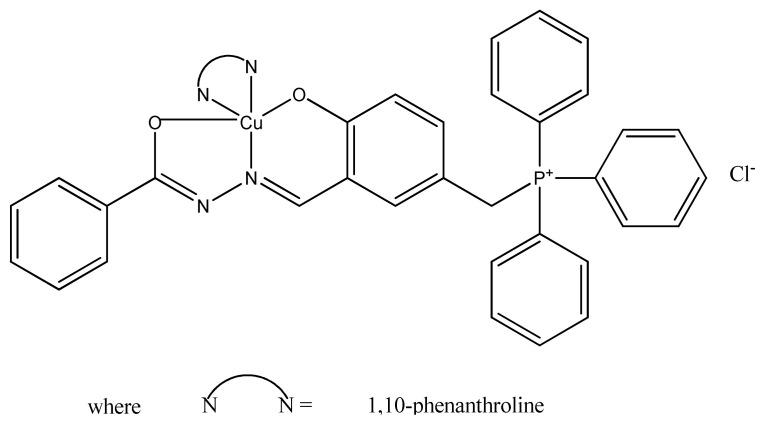
Proposed structure of Cu (II) complex.

Adsule et al. have synthesized copper Schiff base complexes with quinoline-2-carboxaldehyde ligands. The results of copper-quinoline derivatives against prostate cancer cell lines LNCaP and PC-3 showed that they induce apoptosis without causing an oxidative stress and also are less toxic. Additionally, introduction of thiocarbonyl side chains improved the anticancer potency. in fact, Structure 34 (Figure 34) was the most effective analog which showed proteasome inhibitory activity having a IC_50_ value lower than pyrrolidine DTC and clioquinol [144].

Similarly, Cu complexes containing thioxo group such as disulfiram revealed anti-cancerous activity against cancer xenografts or cancer cell lines [145]. It has been demonstrated that mixture of Cu (II) salt with clioquinol and dithiocarbamates (DTCs) actively bind with tumor cells causing a proteasome inhibitor. The anti-angiogenesis effects and proteasome inhibitor in cancer treatment are widely reviewed by several researchers [146,147 ]. Zhong et al. has synthesized Cu (II) (**28**) complex with Schiff base ligand (Z)-2-hydroxy-N’-(2-oxoindolin-3-ylidene) benzohydrazide and characterized by X-ray crystallography as a distorted octahedral shape. The cytotoxicity assays examined against four different cancer cell lines (MGC, SPCA-1, K562 and Tb) indicated that this complex was considerably more active than related compounds formerly reported [ 148,149 ]. Furthermore, the authors claimed that the anti-cancer activity can be improved through intracellular enzymatic reduction into the generation of cytotoxic Cu(I) species [150].

#### 2.1.4. Enzyme inhibition activity

The compounds such as benzenesulfonamide and/or 1,3,4-thiadiazole-2-sulfonamide heads and polyamino-polycarboxylate tails have been used as ligands to prepare some Cu(II) complexes, that were reported to possess potent inhibitory effect against carbonic anhydrase isoform, transmembrane CA IX and XII and cytosolic CA I and II, and could be used as positron emission tomography hypoxia marker of tumor. The inhibitory effect of Cu (II) complexes was stronger as compared with corresponding parent bis-sulfonamides [151]. Four new Schiff base copper (II) complexes copper (II) complexes have been synthesized and characterized. All the complexes were reported to have good inhibitory activity against jack bean urease in vitro with maximum inhibitory effect of complex (**3**), (IC_50_ = 1.45–3.59 μM) [152]. According to another investigation four Schiff bases copper (II) complexes have been found to show excellent inhibitory activities against jack bean urease with maximum inhibitory activity for C_28_H_18_N_6_F_6_O_2_Cu (IC_50_ = 0.49 μM) followed by C_26_H_20_N_6_O_2_Cu (IC_50_ = 1.01 μM), C_26_H_18_N_6_CuF_2_O_2_ (IC_50_ = 0.49 μM) and C_28_H_18_N_6_F_6_O_2_Cu (IC_50_ = 0.49 μM), even much better than standard acetohydroxamic acid (IC50 = 185 μM) [153].

Four copper (II) complexes of hydrazone ligand comprising triphenylphosphonium moieties have been synthesized and studied for their cytotoxicity and topoisomerase I inhibitory effects. Of all the complexes (1,10′ phenanthroline) [5-(triphenyl phosphonium methyl)-salicylaldehyde benzoylhydrazonato] copper (II) monohydrate is shown to exhibits maximum cytotoxic effect against human prostate adenocarcinoma cell line with IC_50_ of 3.2 mM. However, all the four complexes also inhibited topoisomerase I on binding to DNA and enzyme [154]. The copper complexes of two salicylaldehyde derived Schiff bases ligands i.e., 2-{(E)-[(4-chlorophenyl) imino] methyl} phenol and 2-{(E)-[(4-bromophenyl)imino]methyl}phenol were found active against urease enzyme with IC_50_ value of 10.7 and 5μM, respectively as compared to standard inhibitor [155]. The complex 2,6-bis(benzimidazo-2-yl) pyridine copper (II) chloride has been shown to possess metalloprotease activity. It binds to bovine serum albumin causing site specific breakage of the protein when the system is incubated in atmospheric conditions. This is believed to take place through binding and activation of molecular oxygen by the metal [156]. Two new square pyramidal copper(II) complexes *i.e*., [Cu(2,5-pydc)(2-aepy)(H_2_O)]·H_2_O (**1**) and [Cu(2,5-pydc)(2-ampy)(H_2_O)]·H_2_O (**2**) have been investigated for their inhibitory effect against human serum paraoxonase 1 (PON 1) enzyme using diethyl 4-nitrophenyl phosphate as a substrate. Both complexes reduced the PON 1 activity with various inhibition mechanisms in vitro. Inhibitory effect of complex (**1**)is competitive, whereas that of complex(**2**) is noncompetitive [157]. Two mononuclear copper (II) complexes such as [Cu(C_15_H_16_NO_2_)_2_] (**1**) and [Cu(C_6_H_9_N_2_O_4_)_2_·3H_2_O] (2·3H_2_O) have been evaluated for their in vitro inhibitory activities towards *Helicobacter pylori* urease. Both Cu(II) complexes have revealed stronger *H. pylori* urease inhibitory effects with the IC_50_ values of 3.23 μM for (1) and 1.05 μM for (2·3H_2_O), respectively compared with that of acetohydroxamic acid (standard inhibitor)with IC50 of 42.47 μM [158].

**Figure 35:**
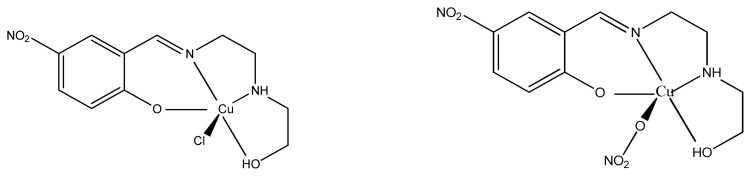
Chemical structures of Cu(II) complex.

A series of new copper(II) complexes with tridentate aroylhydrazone ligands have been synthesized and evaluated for anti-urease activities.Among the series of obtained complexes, [CuClL]·CH_3_OH[L = 4-bromo-N’-(2-hydroxy-5-methoxybenzylidene)benzohydrazide]stands out due its high anti-urease activity (IC_50_ = 0.20 mM) [159]. Two new mononuclear complex of Cu(II) with Schiff base 2-{[2-(2-hydroxyethylamino)ethylimino]methyl}-4-nitrophenol (Figure 35) are investigated for their urease inhibitory activity. Both the complexes showed strong urease inhibitory activities with the values being much lower (IC_50_ = 22.40–24.25 μM)than that of the acetohydroxamic acid (IC50 = 45.32 μM) [160].

**Figure 36:**
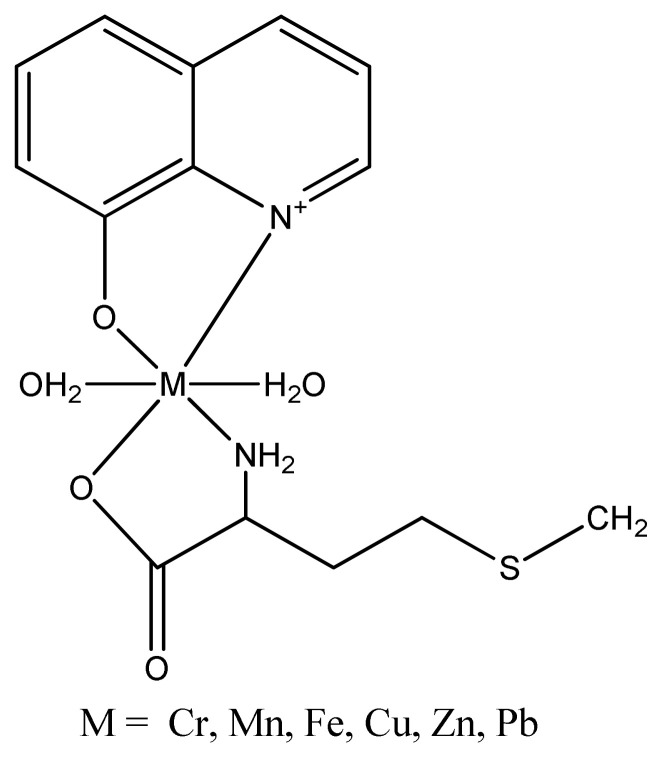
Chemical structure of synthesized Metal (II) complex.

Five complexes of Cu(II) with Schiff bases including [Cu(C_13_H_11_N_2_O)(H_2_O)].ClO_4_, Cu[Cu(CH_3_COO)(C_17_H_16_N_2_O_2_)]_2_, [Cu_2_(C_16_H_24_N_2_O)_2_Cl_4_], [Cu(C_14_H_22_N_2_O)_2_](ClO_4_)_2_, [Cu(C_13_H_11_N_2_O)(H_2_O)](NO_3_).H_2_O have been evaluated for their inhibitory properties on xanthine oxidase (XO).All these complexes indicated good inhibitory activity against XO with IC_50_ values of 96.24, 12.99, 10.38, 23.36 and 81.25 μM, respectively compared with allopurinol (standard inhibitor) with IC_50_ of 10.34 μM [161]. A new Cu (II) complex (Figure 36) synthesized by using 8-hydroxyquinoline and DL-methionine as ligands showed potent inhibitory activity (IC_50_ = 22.6 μM) among all the other complexes when compared with the standard thiourea (IC_50_= 21.6μM) [162].

**Figure 37:**
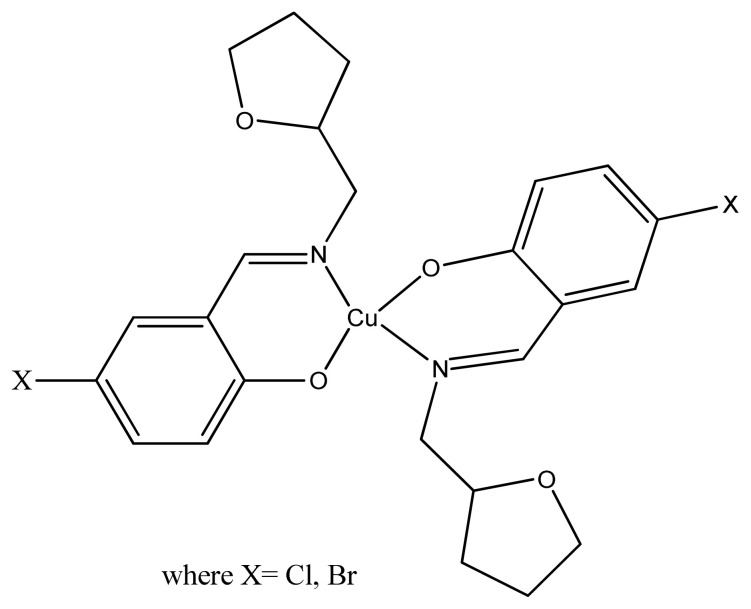
Structure of Schiff base Cu(II) complex.

A new Cu (II) complex synyhesized by using a novel Schiff base ligand 2-(2-hydroxyphenyl)-3-{[(E)-(2-hydroxyphenyl)methylidene]amino}-2,3-dihydroquinazolin-4(1H)-one has been found to exhibit promising activity against urease with an IC_50_ = 0.3 μM and is even more potent than standard thiourea IC_50_ = 0.5 μM [163]. Seven new Schiff base copper(II) complexes (Figure 37) have been prepared. All the complexes showed inhibition of jack bean urease in vitro with IC_50_ range of 2.60–17.00 μM [164].

**Figure 38:**
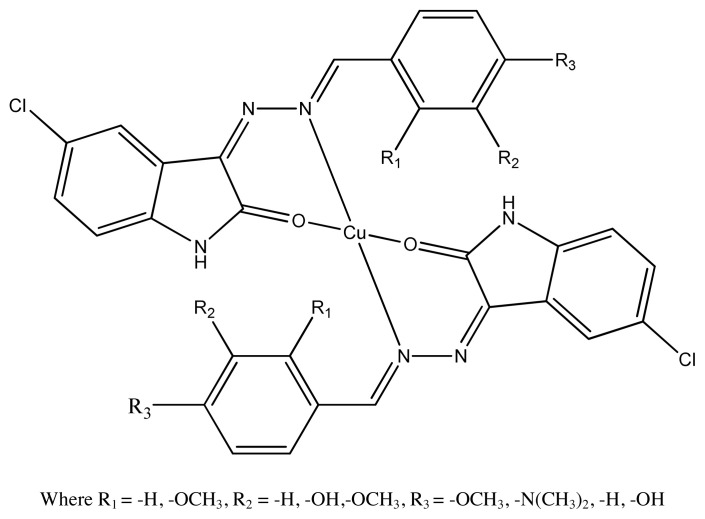
Structure of Cu(II) complexes with bishydrazones.

Six new Copper (II) complexes (Figure 38) with bishydrazone derived from condensation of 5-chloro-isatin monohydrazone with various substituted aromatic aldehydes have been manufactured and screened for urease inhibition activity. The results indicated that the urease inhibitory activity increases with complexation [165].

**Figure 39:**
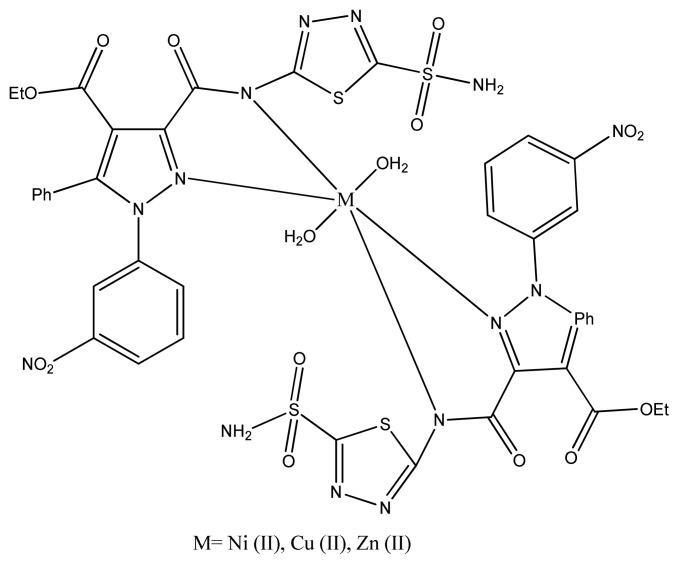
General structure of pyrazole-based sulfonamide complex.

The synthesized isatin-derived bis-Schiff base Cu(II) complexes have been reported to exhibit urease inhibitory activity against Jack bean urease in the range of 8.61 % to 36.4% [166]. Three copper(II) complexes of Schiff base ligand derived from tetrahydrofurfurylamine, have been made and tested in vitro against jack bean urease. The results showed potent inhibitory activities of all three complexes with IC_50_ ranges of 7.20–11.00 μM [167]. A Cu(II) complexe of pyrazole based sulfonamide (Figure 39) has been reported to exhibit potentin vitro inhibitory effect on human erythrocyte carbonic anhydrase isozymes I and II. The complex showed inhibition constant of0.1480 μM for hCA-I and 0.0724 μM for hCA-II, even better than corresponding acetazolamide [168].

Copper(II) ternary complex, [Cu(phen)(C-dmg)(H_2_O)]NO_3_ is not reported to cause significant induction of cytochrome P450 (CYP) 3A and 1A enzymes but inhibited moderately CYP isoforms 1A2, 2C9, 2C19, 2D6, 2B6, 2C8 and 3A4 [169].

Copper(II)metal complex of N-(n-Butyl)ethylenediamine has indicated α-glucosidase inhibition activity with IC_50_ value 1.018 mg/mL [170].

The inhibitory effect of Cu(II) complex was studied on seven CYP isoforms by means of pooled human liver microsome and probe substrate. The complex showed IC_50_ values in the range of 5 to 10 μM against testedCYP isoforms [171]. A novel Cu(II)metal complex with 6-methylpyridine-2-carboxylic acid and2,2′-dipyridylamineas ligand indicate strong α-glucosidase inhibition with IC_50_ = 513.10 [172]. Three copper (II) complexewith 1,6-diaminohexane*i.e*., [Cu(dahe)_3_]_2_Cl, [Cu(dahe)_3_]SO_4_ and [Cu(dahe)_3_]_2_NO_3_ have shown α-glucosidaseinhibition activities with IC_50_ values of 1.332, 1.763 and 1.259 mg/mL [173]. Copper (II) complexe of ethambutol revealed good antioxidants and good inhibition of two enzymes i.e., acetylcholine esterase (IC_50_ = 101μg/mL) and protease (72.78%) [174]. Copper (II) complexes of l,l0-phenanthroline and 2,2’-bipyridine are reported to inhibit strongly against both beef plasma and pig kidney amine oxidase [175].

**Figure 40:**
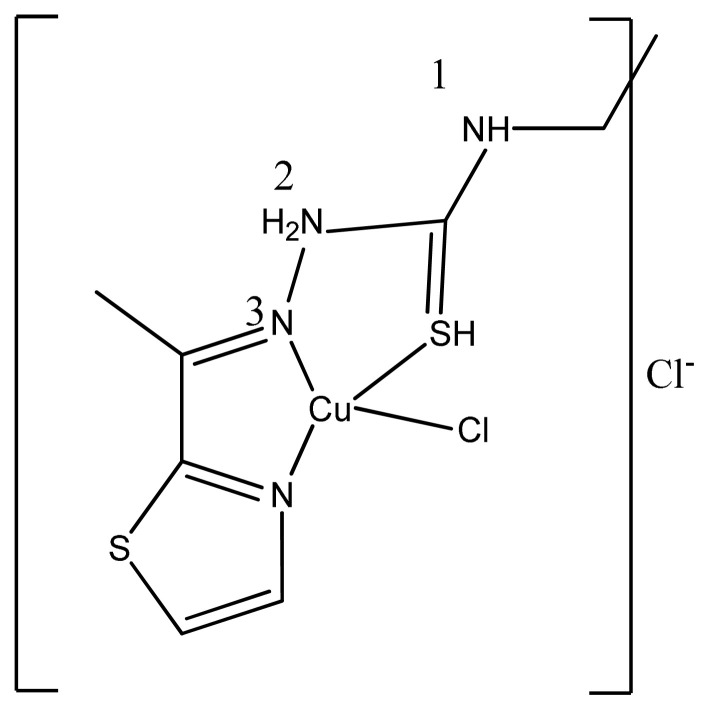
General structure of Cu (II) complex.

A novel copper(II) complex of thiosemicarbazone, (E)-N-ethyl-2-[1-(thiazol-2-yl)ethylidene]hydrazinecarbothioamide (Figure 40) has been reported to act as a poison inhibitor of human topoisomerase IIα, which may also account for the observed anti-cancer effects [ 176].

## 3. Conclusion

In this work, the pharmacological effects of copper metal complexes have been discussed. The bioinorganic chemistry is a growing field because of having connection to medicine. In last few decades, it has been observed that novel copper complexes have therapeutic impact on medicinal field. Advancements in bioinorganic chemistry are necessary to reduce the toxic side effects of ongoing medicines and their mode of action. The attractiveness of such type of compounds is due to binding ability of copper with mononuclear and dinuclear ligands. This review reveals antimicrobial, antioxidant, anticancer, and enzyme inhibition activity of various copper complexes that could be suitable strategies to develop novel diagnostic and therapeutic tools for treatment of various diseases.
